# Auricular Acupressure Versus an Intermittent Low-Carbohydrate Diet in Children With Overweight or Obesity With Gastric-Heat and Dampness-Obstruction Syndrome: Protocol for a Randomized Controlled Trial

**DOI:** 10.2196/59856

**Published:** 2025-03-03

**Authors:** Wen Sun, Jingwei He, Wenqin Wang, Chen Lu, Yating Lin, Yalan Dou, Weili Yan, Jian Yu

**Affiliations:** 1 Department of Traditional Chinese Medicine Children’s Hospital of Fudan University National Children’s Medical Center Shanghai China; 2 Department of Clinical Epidemiology and Clinical Trial Unit Children’s Hospital of Fudan University National Children’s Medical Center Shanghai China

**Keywords:** children with obesity, traditional Chinese medicine, TCM auricular acupressure treatment, intermittent low carbohydrate diet, study protocol

## Abstract

**Background:**

Childhood obesity frequently persists into adulthood and is associated with an increased risk and earlier onset of cardiovascular disease in later life. Behavioral change strategies have been proposed as the first-line weight management approach for children and adolescents with obesity. Nonpharmacological interventions, such as traditional Chinese medicine (TCM) auricular acupressure treatment and intermittent low-carbohydrate diet (ILCD), are increasingly being investigated in the young obese population. However, there is limited high-quality evidence about effectiveness and safety in weight control and reducing cardiometabolic risk in the pediatric population.

**Objective:**

This study aimed to compare the effect of cardiometabolic risk reduction between TCM auricular acupressure treatment (TAAT) and ILCD in children with overweight or obesity with gastric-heat and dampness-obstruction syndrome.

**Methods:**

This is a randomized controlled trial. Eligible participants are children with overweight or obesity and enrolled at the obesity clinic of the department of TCM at a tertiary children’s hospital. Eligible participants must meet the following criteria: (1) be aged between 6 and 18 years, (2) be overweight, and (3) have gastric-heat and dampness-obstruction syndrome. Recruited children will be randomized 3:1 to receive either TAAT or a self-administered ILCD for 1 month: 150 in the TAAT group and 50 in the ILCD group. The primary outcome is the change in body weight from the beginning of treatment to the end of 1 month. Secondary outcomes included body weight, waist circumference, waist-to-height ratio, BMI, blood pressure, body fat content, indexes of liver and renal function, indexes of glucose metabolism, gut microbiota, and TCM syndrome scores at the end of 1 month and 3 months, respectively. Primary statistical analyses were conducted using the intention-to-treat strategy. A generalized linear model was used to compare the difference in weight change between the groups, with the baseline body weight as the covariate, to obtain the estimate of the mean difference in body weight change and its 95% CI, using Gaussian for family function and identity for link function.

**Results:**

The study protocol was approved by the institutional ethical committee and registered on ClinicalTrials.gov on May 5, 2023, before recruitment. Recruitment began in June 2023 and is expected to be completed by December 2025. As of November 2024, we have enrolled 112 participants.

**Conclusions:**

This randomized controlled trial will provide evidence on the treatment effects and safety of TAAT versus ILCD among children with overweight or obesity with gastric-heat and dampness-obstruction syndrome, in reducing body weight and improving cardiometabolic risks. Exploratory aims include potential underlying mechanisms of the 2 kinds of interventions, based on biosamples.

**Trial Registration:**

ClinicalTrials.gov NCT05847478. https://clinicaltrials.gov/study/NCT05847478

**International Registered Report Identifier (IRRID):**

DERR1-10.2196/59856

## Introduction

The Report on Cardiovascular Health and Disease in China (2022) [1] shows that cardiovascular disease is the leading cause of death among urban and rural residents in China, which is mainly caused by hypertension, dyslipidemia, diabetes, overweight or obesity, physical inactivity, inappropriate diet, and metabolic syndrome. It is important to note that obesity, especially abdominal obesity, is a direct risk factor for cardiovascular disease. Unfortunately, obesity can persist from childhood and adolescence into adulthood [2,3]. Obesity in children and adolescents is a global health issue [4], with China, India, the United States, Indonesia, and Brazil being the top-ranked countries, each estimated to have more than one million children with obesity in 2030 [5].

First-line treatments for obesity should be supported by behavioral change strategies [4]. Family-based interventions that address diet, physical activity, sedentary behavior, and sleep quality are recommended. However, the weight loss effect is not ideal and is difficult to maintain. Although evidence for pharmacotherapy and bariatric surgery as supplemental therapies is emerging, they are not recommended for weight loss in children [6]. In fact, safer and more acceptable nonpharmacological interventions, such as traditional Chinese medicine (TCM) and TCM auricular acupressure treatment (TAAT), are increasingly used.

The earliest known mention of TAAT dates back to Huang Di Nei Jing (circa 100 BC) [7,8]. Currently, auricular therapy is widely used in clinical settings for pain relief, epilepsy treatment, improving sleep quality, and obesity [9-11]. This approach offers the advantage of individualized treatment based on the dialectical theory of governance. TAAT has been found effective on children, adolescents, and adult obesity [12-14]. The study found that auricular acupuncture stimulation clearly modulates the feeding-related hypothalamic neuronal activity of experimental (both hypothalamic and dietary) obese rats. The results suggest that auricular acupuncture stimulation may not reduce appetite but is more likely concerned with satiation formation and preservation [15]. However, high-quality clinical evidence is lacking. According to TCM, obesity is located on the spleen and stomach, followed by the liver and kidney. TCM identifies several syndrome types of obesity, including gastric-heat and dampness-obstruction syndrome, spleen-deficiency and dampness-stagnation syndrome, liver-qi stagnation syndrome, and spleen-kidney deficiency syndrome [16,17]. Among these, gastric-heat and dampness-obstruction syndrome is the most common type [18]. In order to ensure the sample size is attained, the gastric-heat and dampness-obstruction syndrome has been selected for inclusion in the study. In addition, the choice of a single syndrome in preference to multiple options ensures comparability in the evaluation of efficacy between groups.

Numerous studies have demonstrated the effectiveness of low carbohydrate diets (<50 g per day or <10% energy from carbohydrates) in combating obesity [19], deemed feasible and acceptable [20,21]. Our previous research has shown that the intermittent low carbohydrate diet (ILCD) was effective in improving weight-related outcomes in children and adolescents [22]. However, the efficacy needs to be further investigated, and long-term effects are uncertain. So far, there are currently no randomized controlled trials comparing the effectiveness between ILCD and TAAT. At the same time, our secondary aim is to explore the mechanistic differences between the 2 nondrug interventions. Therefore, this study aims to assess whether TAAT as the experimental arm is more effective than ILCD as the control arm in improving the cardiometabolic risk in children with overweight or obesity with gastric-heat and dampness-obstruction syndrome.

## Methods

### Objective and Trial Design

This is a randomized controlled superiority trial that aims to compare the effects of TAAT versus ILCD in reducing the cardiometabolic risk in children with overweight or obesity with gastric-heat and dampness-obstruction syndrome. Participants were allocated in a 3:1 ratio to either an auricular acupressure treatment group or a control group. The TCM clinicians were aware of the allocation but did not participate in the following research. Conversely, the outcome observers were blinded about the allocation.

### Recruitment and Study Setting

Eligible participants were recruited by a pediatrician at the obesity clinic of the Department of TCM in Children’s Hospital of Fudan University. All participants were required to provide written consent before intervention.

### Participants Eligibility Criteria

Eligible participants must meet the following criteria: (1) be aged between 6 and 18 years, (2) be overweight [23], and (3) have gastric-heat and dampness-obstruction syndrome [16].

Overweight or obesity with gastric-heat and dampness-obstruction syndrome was defined as follows:

(1) The “Expert Consensus on Diagnosis, Assessment, and Management of Obesity in Chinese Children” states that BMIs between 85% and 95% were classified as overweight, while those above 95% were classified as obese for children of the same gender and age [23].

(2) The “Pediatrics of Traditional Chinese Medicine” framework [16] outlined the conditions that must be met for the gastric-heat and dampness-obstruction syndrome, which include (1) dizziness and heaviness in the head, (2) thirst, (3) rapid digestion of food and polyorexia, (4) heaviness of limbs, (5) fatigue body and lack of strength, (6) a red tongue, (7) greasy fur, and (8) a slippery pulse. It is important to note that conditions (7) and (8) must be satisfied, while conditions (1) to (6) must be satisfied at least 3 conditions.

Participants who met any of the following exclusion criteria were excluded: (1) having hereditary obesity or obesity caused by endocrine disorders, such as Cushing syndrome, primary hypothyroidism, and hypothalamic obesity; (2) currently being enrolled in other clinical trials or having participated in another clinical trial in the past 3 months; (3) self-administration of oral weight-loss medications or other forms of meal replacements; and (4) having serious organic diseases of the heart, liver, kidneys, or brain, or infectious diseases or psychiatric disorders.

### Interventions

#### Experimental Group (TAAT Group)

Specialized TCM clinicians conducted TAAT following a standardized procedure. The location and operation of the ear acupoints followed the guidelines outlined in the “National Standard of the Peoples Republic of China for Nomenclature and Location of Auricular Points (GB/*t* 13734-2008).” In addition, all TCM clinicians had received specialized training to ensure a comprehensive understanding of the auricular acupressure intervention program and to standardize the procedures performed by different clinicians.

**Figure 1:**
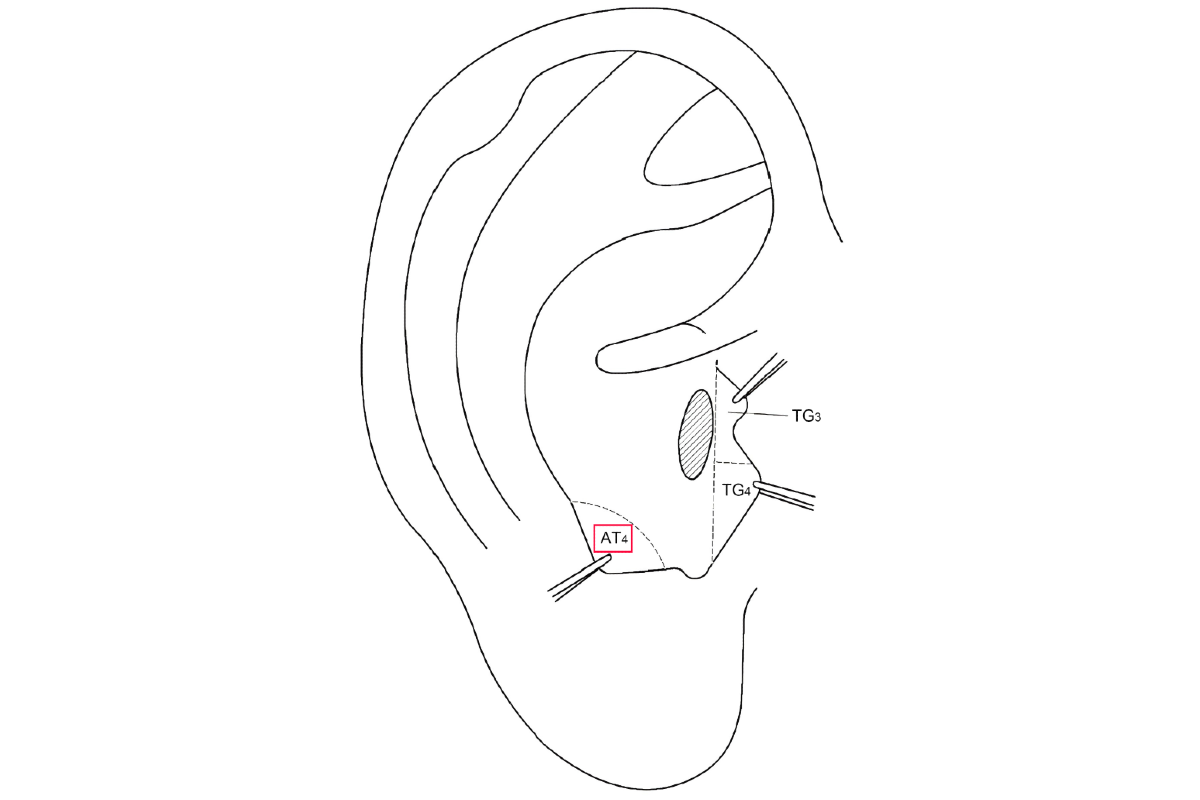
Location of the subcortex (AT4).

**Figure 2:**
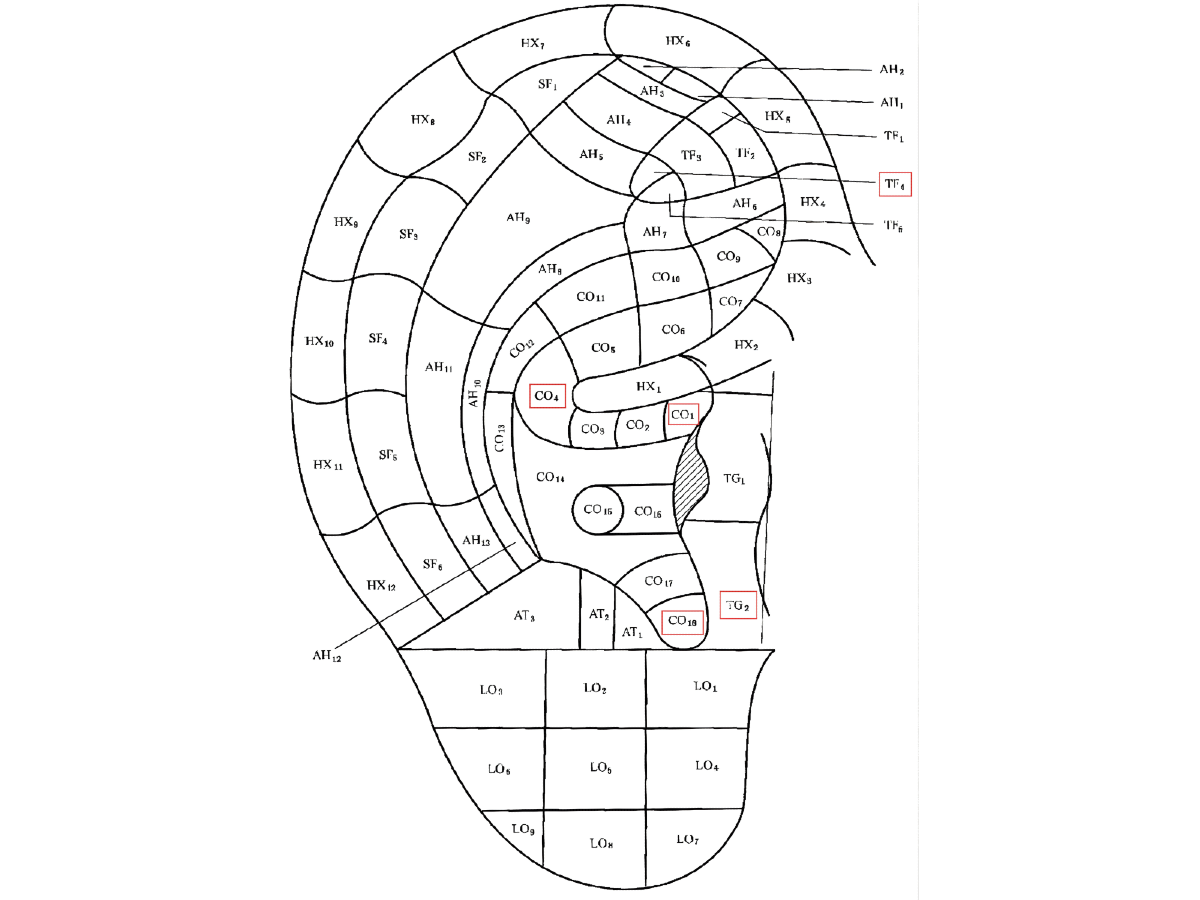
Location of the shenmen (TF4), Xiaoping (TG2), endocrine (CO18), stomach (CO4), mouth (CO1).

The study selected the following ear acupoints for acupressure treatment: Shenmen (TF4), Xiaoping (TG2), Endocrine (CO18), Stomach (CO4), Mouth (CO1), and Subcortex (AT4) (refer to Figures 1 and 2) [24]. The acupressure was administered using an opaque patch with Vaccaria seed. Participants were instructed to apply pressure for 1 minute before and after meals each day, for a total of 2 to 3 days per week. They were followed weekly and received a 3-course treatment, with 1 course per month.

#### Control Group (ILCD Group)

Participants were required to limit their daily carbohydrate intake to 50 g or less, while their dietary calorie intake was not restricted for nonconsecutive 7 days of 2 weeks, corresponding to 14 days in a month. On the remaining days, participants should maintain their normal diet. The intervention period lasted for 1 month, followed by a 2-month follow-up period [20].

### Follow-Up Period

First, a WeChat group was created for parents of participants to facilitate communication and monitoring about diet and exercise recording, as well as scheduling follow-up appointments. Second, adherence to the study interventions were uniformly assessed in both groups at each visit. Third, if participants missed any visits, investigators would contact them directly by telephone to encourage continued participation and evaluate barriers.

The research assistant attempted to establish contact with the missed participants. A dropout participant was defined as someone who had not been reached for 3 months. If participants decided to withdraw before completion, all collected data would be remained for analysis.

### Outcomes

#### Primary Outcome

Change in body weight from the beginning of the treatment to 1 month.

#### Secondary Outcomes

Secondary outcomes included body weight, waist circumference, waist-to-height ratio, BMI, blood pressure (BP), body fat content, glucose metabolism, blood lipid levels, liver and kidney function, composition of gut microbiota, and TCM syndrome score, and were assessed at the end of 1 month and 3 months, respectively.

### Measurement

#### Anthropometry

Including the measurements of weight, height, waist circumference (WC), and BP. These measurements should be taken by the same person, using the same instruments, at the same time of day. Each measurement should be taken 3 times in a row, and the average of the 3 values should be used.

(1) **Weight measurement:** The participant should be weighed in light clothing and preferably with an empty bladder. They should also remove shoes, heavy jewelry, and watches. The electronic weight scale should display 0.0 before the participant stands on it. The participants should stand with their feet together in the center and their heels against the back edge of the scales. The participant’s arms should be hanging loosely at their sides and their head should be facing forward. The weight measurements were recorded in kilograms and were accurate to 0.1.

(2) **Height measurement:** Height should be measured using a stadiometer with the participant standing barefoot and with feet together. The head should be positioned level with a horizontal Frankfurt plane, which is an imaginary line from the lower border of the eye orbit to the auditory meatus. Any hair knots or braids that may interfere with the measurement should be untangled. The press plate should be in contact with the head with moderate tightness. The height measurements were recorded in centimeters with an accuracy of 0.1.

BMI was a widely used tool for assessing the risk of overweight and obesity in individuals. It was calculated using the following equation:



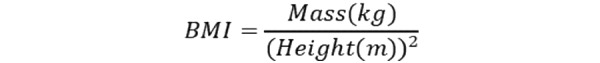



(3) **Waist circumference measurement:** The measurement should be taken while the participant exhales. WC was measured by placing a tape measure around the waist, 1 cm below the navel, with the participant standing and arms naturally at their sides. Record the data in centimeters with an accuracy of 0.1.

Waist-to-height ratio had emerged as a surrogate for abdominal obesity that also takes into account body size and allows the same cut of across age and gender [25]. It was calculated using the following equation:



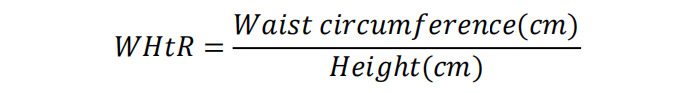



(4) **Blood pressure measurement:** BP was measured on the right arm using the CONTEC08A electronic sphygmomanometer (CONTEC Medical Systems Co). The participant should be seated in a quiet room for 3-5 minutes before measurement, with the back supported and feet uncrossed on the floor. The right arm should be at heart level, supported, and uncovered above the cuff. The correct cuff size should be used. The bladder length should be 80%-100% of the circumference of the arm, and the width should be at least 40%.

#### Body Fat Content

Body composition was measured by determining the body fat percentage using the LUNAR dual photon X-ray dual-energy X-ray absorptiometry equipment (General Electric Company). Dual-energy X-ray absorptiometry was a medical imaging technology that used low dose radiation for a whole-body scan to provide accurate measurements of fat percentage. The equipment was operated by qualified professionals.

#### Venous Blood Tests

The tests were conducted after a 10-hour overnight fast to avoid any influence of diet on the test results. The tests included glucose metabolism, blood lipid levels, and liver and kidney function. The remaining blood samples were stored in a –80℃ refrigerator for further testing. The operators conducting the tests possessed professional qualifications.

#### TCM Syndrome Score

“Pediatrics of Traditional Chinese Medicine” listed a number of TCM symptoms in obesity with gastric-heat and dampness-obstruction syndrome: (1) dizziness and heaviness in the head, (2) thirst, (3) rapid digestion of food and polyorexia, (4) heaviness of limbs, (5) fatigue body and lack of strength, (6) a red tongue, (7) greasy fur, and (8) a slippery pulse. Each item was scored on a scale of 0-2, with 2 points for obvious symptoms, 0 points for nonsymptomatic, and 1 point for symptoms that are between obvious and nonsymptomatic. The total syndrome score was obtained by adding up the scores for each item. A qualified clinician performed the score evaluation.

#### Gut Microbiota Test

Stool samples were collected using Boyou nucleic acid storage tubes (Shanghai Biotechnology Corporation) and stored in a laboratory refrigerator at –80℃. To study intestinal flora diversity, the 16S rDNA target region would be amplified, and information on intestinal microbial diversity and community composition will be obtained by detecting sequence variation and abundance of the target region. The operators possessed professional qualifications.

#### Dietary and Exercise Data

Diet and exercise data were recorded by the children and their families, but they were educated by qualified clinicians before recording [26,27].

(1) **From the baseline to the first month:** The weight of the food was measured using an electronic scale before and after each meal, and photographs were taken as well. Participants were required to wear sports watches every day, including during sleep, but not while bathing, swimming, or in other special cases. Dietary data included the type and weight of the foods consumed, while exercise data included step count, calorie consumption, resting heart rate, sleep duration, and exercise type. All data was meticulously recorded daily in the “Diet and Exercise Handbook.”

(2) **From the second month to the third month:** The method and content remained unchanged. Dietary and exercise data were recorded on Thursdays, Fridays, and Saturdays during 1 week, and distributed at the beginning, middle, and end of the month, rather than every day.

### Participant Timeline

The participant timeline was shown in Table 1, following the Standard Protocol Items: Recommendations for Interventional Trials (SPIRIT) diagram [28].

**Table 1:** Schedule of enrollment, interventions, and assessments for the trial. t1, preinclusion assessment; t0, baseline; t1, intervention for one 1 month; t2, intervention for 3 months.

Time point					Enrolment (–t_1_)		Allocation (t0; Day 0)		Post allocation					Closed out
									*t*_1_ *1 month*		*t*_2_ *3 months*			
**Enrolment**														
			Eligibility screen		✔		N/A^a^		N/A		N/A		N/A	
			Informed consent		✔		N/A		N/A		N/A		N/A	
			Allocation		N/A		✔		N/A		N/A		N/A	
**Interventions**														
		Auricular acupressure		N/A		N/A		✔		✔		N/A		
		Intermittent carbohydrate restriction diet		N/A		N/A		✔		N/A		N/A		
**Assessments**														
	General investigation			N/A		✔		✔		✔		N/A		
	Anthropometry (Height, Weight, WC^b^, and BP^c^)			N/A		✔		✔		✔		N/A		
	Body fat content			N/A		✔		✔		✔		N/A		
	Indexes of liver and renal functions			N/A		✔		✔		✔		N/A		
	Indexes of glucose metabolism			N/A		✔		✔		✔		N/A		
	Levels of serum lipid			N/A		✔		✔		✔		N/A		
	TCM^d^ syndrome score			N/A		✔		✔		✔		N/A		
	Gut microbiota			N/A		✔		✔		N/A		N/A		
	Dietary data			N/A		✔		✔		✔		N/A		
	Exercise data			N/A		✔		✔		✔		N/A		
	Adverse events			N/A		N/A		✔		✔		N/A		

^a^N/A: not applicable.

^b^WC: waist circumference.

^c^BP: blood pressure.

^d^TCM: Traditional Chinese Medicine.

### Sample Size

The primary outcome was the change in body weight from baseline to 1 month. The sample size calculation was based on the difference in weight change between the 2 groups: the TAAT group and the ILCD group.

Our pilot data from 12 patients revealed a weight loss of 1.9 kg after 4 weeks of TAAT and a weight reduction of 1.4 kg after 4 weeks of ILCD, with an SD of 1.1 kg. We assumed a 0.5 kg difference in weight reduction between the 2 arms, power of 0.80, a 2-sided α of .05, and a sample size ratio of 3:1, a minimum sample size of 150 for the TAAT group and 50 for the ILCD group were required, respectively. Assuming a dropout rate of 15%, the trial enrolled at least 240 participants. The “test for 2 means module” of the PASS 16.0 software was used.

### Recruitment

The estimated period for recruitment, intervention, and data collection would take probably 30 months to complete. Eligible clients attending the obesity clinic at the Children’s Hospital of Fudan University were invited to participate in the study through collaboration with TCM clinicians. The time required for participant recruitment and baseline assessment was approximately 30 to 45 minutes.

### Randomization and Blinding

Participants were randomly assigned to either the experimental or control group through a simple randomization process generated by the R Language (version 4.2.2; R core team). This process was designed by the clinical trial unit of the Children’s Hospital of Fudan University and follows a 3:1 allocation ratio.

The study coordinator, who was not involved in data collection, performed the allocation. The randomization list was kept strictly confidential. Allocation was done by the study coordinator in a sequentially numbered fashion using identical, opaque, sealed envelopes. The recruiting clinicians were blinded to study group allocation and did not have access to the allocation sequence.

Eligible participants were enrolled by trained clinicians. The study coordinator independently assigned participants to either the intervention or control groups. Outcome observers were blinded. The analysis team received aggregated data obtained for the control and experimental groups. Study participants could be blinded, but they were scheduled for separate follow-up dates.

### Adverse Events and Assessment of Safety

After participants provided informed consent and enrolled in the study, any adverse events were collected and recorded until the end of the study period. Adverse events that occurred after consent was signed, but before receiving the study intervention was reported as not related to our intervention. Any serious adverse event that occurred would be reported to the principal investigator and documented.

### Data Management and Monitoring

Trained clinicians were input data onto a paper-based clinical research form designed by the research team. Once data collection was complete for each participant, it would be entered into an electronic database with built-in checks to ensure completeness. Data monitoring has occurred periodically throughout the study. We used access to create a database, and 2 investigators independently performed dual data entry twice. Once verified, the database was saved appropriately. After confirming the accuracy of the database, it was locked and submitted to an independent statistical team for analysis.

### Statistical Analysis

Primary statistical analyses were conducted using the intention-to-treat strategy. Sensitivity analyses were performed using per-protocol set analysis.

#### Analysis of Primary Outcome

The primary outcome in this study was the change in body weight from baseline to 1 month. A generalized linear model (GLM) was used to compare the difference in weight change between the groups, with the baseline body weight as the covariate, to obtain the estimate of the mean difference in body weight change and its 95% CI, using Gaussian for family function and identity for link function.

#### Analysis of Secondary Outcomes

GLM was used to obtain differences in effect sizes and their 95% CI for continuous variables between groups. Categorical variables were described in absolute numbers (percentages) and GLM was used to obtain differences in effect values and their 95% CI, using logistic for family function and logit for link function.

Statistical analyses were conducted using Stata 16.0 software (StataCrop). All statistical tests were 2-sided with a significance level of α=.05. Differences were considered statistically significant at *P*<.05.

### Ethical Considerations

This study has been approved by the Medical Ethics Committee of the Children’s Hospital of Fudan University and will be conducted in accordance with the approved guidelines and regulations of the participating institution (approval number 2021 [.350]). This trial was conducted in accordance with the current version (AP_IDR_2.0) of the protocol, which has been registered at ClinicalTrials.gov. Informed written consent was obtained from all patients before inclusion in the study. As the involvement of the patients were voluntary, the study could be stopped at any time. All records that contain names or other personal identifiers were stored separately from study records identified by code number. The identification of participants in any images of the manuscript or supplementary material was impossible. For example, the researchers provided the necessary recording materials and sample collectors. The intervention method did not entail additional costs for the participants; thus, no compensation was required.

## Results

**Figure 3:**
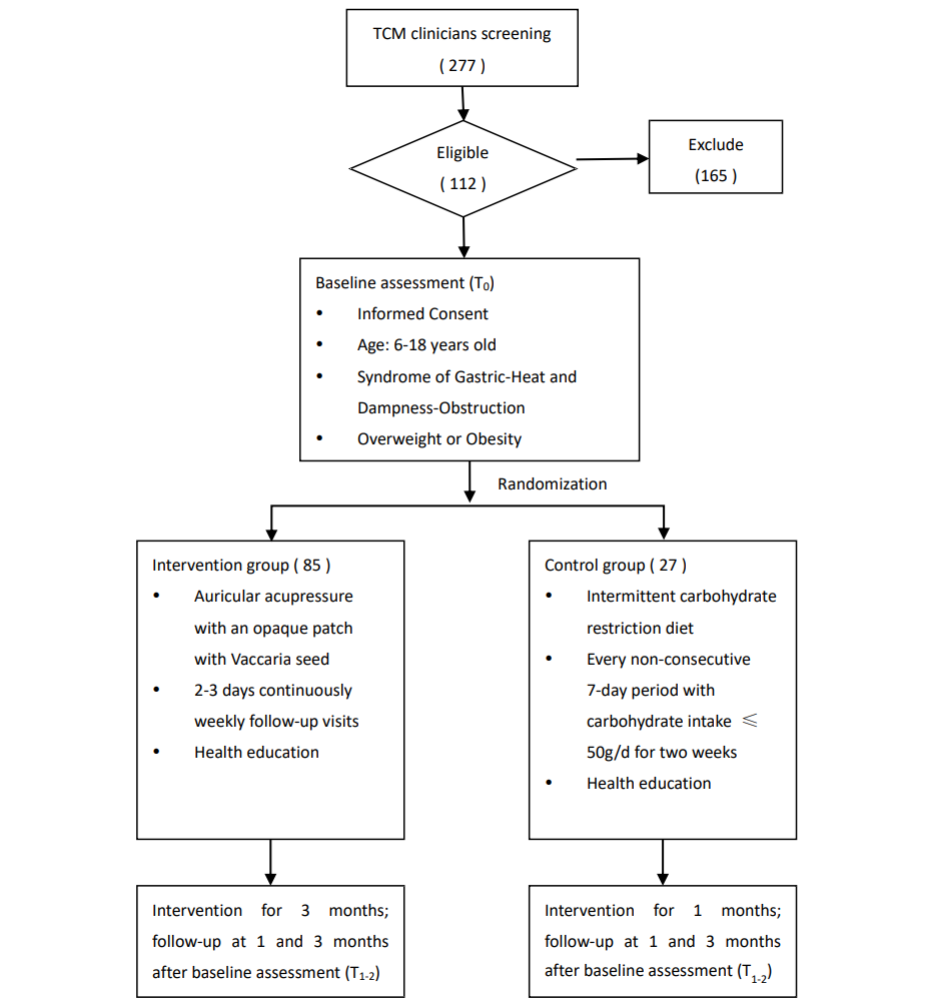
Flowchart of the study. TCM: traditional Chinese medicine; T0: baseline; T1: intervention for 1 month; T2: intervention for 3 months.

The protocol was registered on ClinicalTrials.gov (NCT05847478) on May 5, 2023. The current protocol is version 2.0, dated February 28, 2023, which is the final version as approved by the institutional ethical committee. Recruitment started in June 2023 and is expected to be completed in December 2025. By November 2024, we had enrolled 112 participants (Figure 3).

## Discussion

### Anticipated Findings

It is hypothesized that TAAT will prove superior to ILCD in controlling body weight through this clinical trial. In addition, it is anticipated that the mechanistic differences between the 2 nondrug intervention methods will be elucidated. This study will be the first randomized controlled trial to investigate the effect and safety of TAAT compared with ILCD for overweight or obesity and other cardiometabolic risk factors. Previous studies on the effects of auricular treatment have mainly focused on adolescents and adults [12,14]. A study was conducted as a randomized controlled trial to examine the effects of auricular acupressure on obesity improvement in children. However, this trial had a small sample size and did not take into account the impact of diet, exercise, and lifestyle on body weight [15]. Consequently, there is a lack of high-quality clinical trials on the effects of TAAT in children with obesity, although we found some clinical evidence of this approach in children. Furthermore, the participants’ gut microbiota will be studied in order to explore the underlying mechanisms of the 2 different interventions.

All participants in this study are patients from the Department of TCM and seeking TCM intervention. As one of the treatment methods in TCM, previous studies have shown that TAAT is effective in reducing body weight, WC, and body fat mass [14,29]; therefore, we did not consider a blank control group, instead, using an ILCD, according to its effect indicated in recent randomized trials [30,31].

TAAT appears minimal side effects, including the pain caused by local noninvasive stimulation that can be tolerated by most children [32]. As participants are recruited from the obesity clinic of the Department of TCM, randomization is set in a 3:1 ratio, allowing that needs of more participants are satisfied.

Maintaining compliance remains challenging to the young population. To ensure better compliance, we use methods such as regular reminders and random checks. To avoid bias, the outcome observer is unaware of the grouping of the participants.

In conclusion, we will evaluate the efficacy and safety of the TAAT in losing weight and improving the cardiometabolic risk of overweight or obese children with gastric-heat and dampness-obstruction syndrome. The results of this study will lead to an update of the consensus on TCM intervention for obesity. However, given the limitations of the intervention time in the experimental design, it is hoped that in future clinical trials, the intervention and follow-up time will be extended in order to obtain a long-term evaluation of the intervention effect.

### Conclusion

We conducted a pragmatic randomized controlled trial to investigate the effect and safety of TAAT compared with ILCD for overweight or obese children comorbid with at least one cardiometabolic risk factor. Furthermore, our study will investigate the participants’ differential responses of gut microbiota and serum biomarkers to examine underlying mechanisms. The protocol of our study will provide methodologic information and obtain interesting evidence from children for the 2 kinds of nonpharmacological interventions.

## Appendix

Multimedia Appendix 1 SPIRIT (Standard Protocol Items: Recommendations for Interventional Trials) checklist.

Multimedia Appendix 2 CONSORT (Consolidated Standards of Reporting Trials) checklist.

## Abbreviations

BP: blood pressure
GLM: generalized linear model
ILCD: intermittent low carbohydrate diet
SPIRIT: Standard Protocol Items: Recommendations for Interventional Trials
TAAT: TCM auricular acupressure treatment
TCM: traditional Chinese medicine
WC: waist circumference

## Data Availability

The datasets analyzed in this study are available from the corresponding author upon reasonable request.
