# Correction: Auricular Acupressure Versus an Intermittent Low-Carbohydrate Diet in Children With Overweight or Obesity With Gastric-Heat and Dampness-Obstruction Syndrome: Protocol for a Randomized Controlled Trial

**DOI:** 10.2196/74014

**Published:** 2025-03-28

**Authors:** Wen Sun, Jingwei He, Wenqin Wang, Chen Lu, Yating Lin, Yalan Dou, Weili Yan, Jian Yu

**Affiliations:** 1 Department of Traditional Chinese Medicine Children’s Hospital of Fudan University National Children’s Medical Center Shanghai China; 2 Department of Clinical Epidemiology and Clinical Trial Unit Children’s Hospital of Fudan University National Children’s Medical Center Shanghai China

In “Auricular Acupressure Versus an Intermittent Low-Carbohydrate Diet in Children With Overweight or Obesity With Gastric-Heat and Dampness-Obstruction Syndrome: Protocol for a Randomized Controlled Trial” the authors noted 3 changes.

The author affiliation information has been changed from:

Wen Sun^1^, MSc; Jingwei He^1^, MSc; Wenqin Wang^1^, MSc; Chen Lu^1^, PhD; Yating Lin^1^, BSc; Yalan Dou^2^, PhD; Weili Yan^2^, PhD; Jian Yu^1^, PhD

^1^Department of Rehabilitation, Children’s Hospital of Fudan University, National Children’s Medical Center, Shanghai, China.

^2^Department of Clinical Epidemiology and Clinical Trial Unit, National Children’s Medical Center, Children’s Hospital of Fudan University, Shanghai, China.

to:

Wen Sun^1*^, MSc; Jingwei He^1*^, MSc; Wenqin Wang^1^, MSc; Chen Lu^1^, PhD; Yating Lin^1^, BSc; Yalan Dou^2^, PhD; Weili Yan^2^, PhD; Jian Yu^1^, PhD

^1^Department of Traditional Chinese Medicine, Children’s Hospital of Fudan University, National Children’s Medical Center, Shanghai, China.

^2^Department of Clinical Epidemiology and Clinical Trial Unit, Children’s Hospital of Fudan University, National Children’s Medical Center, Shanghai, China.

^*^these authors contributed equally

An equal contribution footnote has also been added for authors Wen Sun and Jingwei He.

In addition, under “Participant Timeline in Methods”, in Table 1, the entries for Auricular acupressure at *t*_1_ 1 month and *t*_2_ 3 months, and Intermittent carbohydrate restriction diet at *t*_1_ 1 month have been changed from:

“N/A”

to:

“✓”.

We intend to convey that the intervention duration for auricular acupressure is 3 months, while the intervention duration for dietary restriction is 1 month. It appeared incorrectly in the final published version.

The correction will appear in the online version of the paper on the JMIR Publications website, together with the publication of this correction notice. Because this was made after submission to PubMed, PubMed Central, and other full-text repositories, the corrected article has also been resubmitted to those repositories.

